# Circulating microRNA as a Biomarker for Coronary Artery Disease

**DOI:** 10.3390/biom10101354

**Published:** 2020-09-23

**Authors:** Ibrahim T. Fazmin, Zakaria Achercouk, Charlotte E. Edling, Asri Said, Kamalan Jeevaratnam

**Affiliations:** 1 Faculty of Health and Medical Science, University of Surrey, Guildford GU2 7AL, UK; itf21@cam.ac.uk (I.T.F.); za270@cam.ac.uk (Z.A.); c.edling@surrey.ac.uk (C.E.E.); 2 School of Clinical Medicine, University of Cambridge, Cambridge CB2 1TN, UK; 3 School of Medicine, University Malaysia Sarawak, Kota Samarahan 94300, Sarawak, Malaysia; sasri@unimas.my

**Keywords:** coronary artery disease, biomarkers, noncoding RNA, microRNA

## Abstract

Coronary artery disease (CAD) is the leading cause of sudden cardiac death in adults, and new methods of predicting disease and risk-stratifying patients will help guide intervention in order to reduce this burden. Current CAD detection involves multiple modalities, but the consideration of other biomarkers will help improve reliability. The aim of this narrative review is to help researchers and clinicians appreciate the growing relevance of miRNA in CAD and its potential as a biomarker, and also to suggest useful miRNA that may be targets for future study. We sourced information from several databases, namely PubMed, Scopus, and Google Scholar, when collating evidentiary information. MicroRNAs (miRNA) are short, noncoding RNAs that are relevant in cardiovascular physiology and pathophysiology, playing roles in cardiac hypertrophy, maintenance of vascular tone, and responses to vascular injury. CAD is associated with changes in miRNA expression profiles, and so are its risk factors, such as abnormal lipid metabolism and inflammation. Thus, they may potentially be biomarkers of CAD. Nevertheless, there are limitations in using miRNA. These include cost and the presence of several confounding factors that may affect miRNA profiles. Furthermore, there is difficulty in the normalisation of miRNA values between published studies, due to pre-analytical variations in samples.

## 1. Introduction

Coronary artery disease (CAD) is a significant cause of morbidity and mortality in the elderly. It is a complex, chronic pathological process in the intima of coronary arteries, yielding atherosclerotic lesions that restrict blood flow to the myocardium and may be associated with a degree of inflammation. Whilst the disease can remain stable, acute plaque rupture followed by coronary artery thrombosis can be a fatal event. Early detection of this disease will allow for early management and intervention, reducing morbidity and mortality.

Biomarkers are defined as characteristics that may be measured as indicators of normal biological processes or pathogenic processes [1]. Biomarkers may involve several modalities, such as substances measured in the blood and other bodily fluids, as well as imaging results and technologies like electrocardiography; in particular, multi-biomarker approaches may be promising approaches for the better detection of pathophysiology [2]. Currently, CAD detection involves several modalities. Functional tests, such as stress electrocardiograms, and anatomical imaging, such as angiography, provide clinicians with indications of CAD severity [3]. Numerous studies have assessed the validity of these modalities. Their reliability, whilst being generally suitable, varies depending on context [4]. A potential reason for this is variations between heterogenous study populations; however, simultaneous consideration of different biomarkers may improve reliability [4].

Recently, microRNAs (miRNA) have been proposed as a potential biomarker for use in various clinical contexts. They are major effectors of gene silencing through post-transcriptional repression and mRNA degradation [5]. This review aims to discuss the potential utility of microRNA (miRNA), as a diagnostic and prognostic tool for clinicians to detect CAD.

## 2. Localisation of miRNA

**Figure 1:**
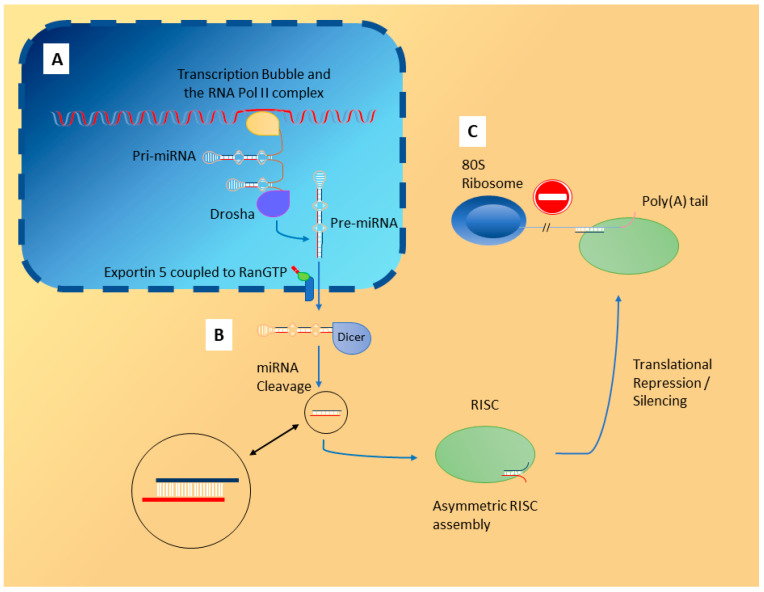
MiRNA biogenesis and their means of transcriptional silencing. RNA Pol II: RNA polymerase II; miRNA: microRNA; RanGTP: Ran coupled to guanosine triphosphate; RISC: RNA-induced silencing complex; Poly(A) tail: poly-adenosine tail; 80S ribosome: eukaryotic ribosome. (**A**) Within the nucleus (blue area), miRNA are initially transcribed (e.g., from an miRNA gene) from DNA by RNA polymerase II (yellow) in the form of primary miRNA, or pri-miRNA, which contain stem-loop structures. The enzyme Drosha (purple) proceeds to cleave these stem–loop structures from the rest of the transcript, and these structures are now defined as precursor miRNA, or pre-miRNA. These are then exported from the nucleus via exportin 5 coupled to the Ran cycle. (**B**) Once in the cytosol (yellow area), the enzyme Dicer recognises pre-miRNA and cleaves them to produce mature miRNA molecules with two nucleotide overhangs on their 3′ ends. This molecule is then incorporated into an RNA-induced silencing complex (RISC, green) and the passenger strand (red backbone) is destroyed. This results in an active RISC complex. (**C**) The active RISC complex uses the guide strand of the miRNA (blue backbone) to target mRNA transcripts, specifically those that are complementary to the seed sequence of the guide strand. Through translational repression and RNA decay, miRNA reduce the expression of certain genes through RISC. Also note that the poly(A) tail is shown in pink. Ago2: Argonaute 2; DGCR8: DiGeorge syndrome critical region 8.

MiRNA are short RNAs (18–25 nts) that engage in the sequence specific inactivation of mRNA (Figure 1). They are encoded by their own non-protein coding genes located across the genome, though they also occur in the introns and exons of other genes [6,7]. MiRNAs are predominantly located intracellularly, although a proportion of them can be detected in the extracellular environment (ECmiRNA), including in plasma and various other body fluids [8,9,10]. They occur freely circulating or associated with other molecules, including within extracellular vesicles, such as exosomes and microvesicles, and can also be complexed with lipoproteins [11,12,13,14,15].

**Figure 2:**
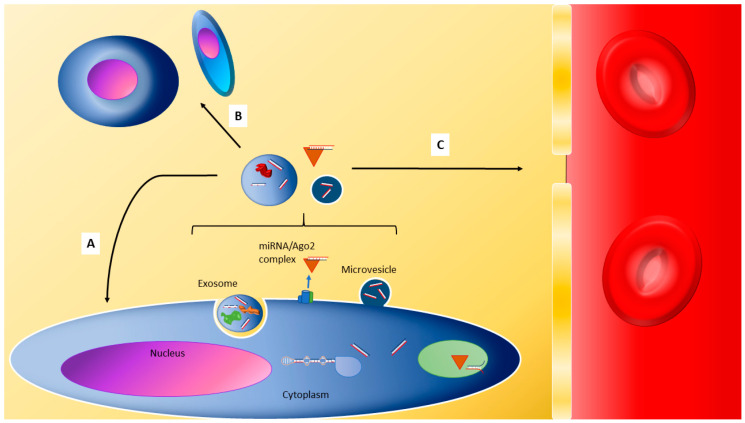
Export pathways for miRNA and means of interaction with other cells/cell of origin as a potential means of signalling. (**A**) The autocrine pathway, whereby extracellular miRNAs re-enter the cell from which they originated. (**B**) The paracrine pathway, whereby extracellular miRNAs are transported towards and enter cells of the same or different type to the miRNA’s cell of origin. (**C**) The endocrine pathway, whereby extracellular miRNAs enter the circulation and are thus transported to cells in other tissues/organs. Ago2: Argonaute 2; miRNA: microRNA.

Microvesicles and exosomes are both types of extracellular vesicles with multiple roles in normal cell physiology. One of their major functions is intercellular communication through the carriage of signalling molecules, including proteins, mRNAs, and miRNAs amongst others, to targets of variable distance from the cell of origin [16,17]. Exosomes have a size range of 30–100 nm, and themselves originate from organelles of the endocytic pathway, the multivesicular bodies [16,17]. A multivesicular body is produced by the invagination of an endosome to produce intraluminal vesicles, into which specific molecules are sorted. Multivesicular bodies are trafficked to and subsequently fuse with the plasma membrane, at which point the intraluminal vesicles, now labelled as exosomes, are released into the extracellular space [16,17]. Microvesicles have a size range of 0.1–1.0 μm and are produced from the plasma membrane directly through outward blebbing [17,18]. Specific molecular cargo is transported towards regions of the plasma membrane where local alterations in the lipid composition reduce the rigidity of the membrane and facilitate further curvature [17,18]. The assembly of contractile machinery in these regions produce cytoskeletal rearrangements that pinch off nascent microvesicles [17,18]. The membrane budding that produces microvesicles differs from the blebbing process that produces apoptotic bodies, which is a less specific process [17,18]. These extracellular vesicles can then be trafficked through autocrine, paracrine, and endocrine paths (Figure 2). The multiple forms of endocytosis are the typical forms of vesicle uptake, though membrane fusion between microvesicles and the target cell plasma membrane has also been observed [17]. The mechanism utilised is likely dependent on the recipient cell type and the suitable expression of receptors compatible with the vesicle [17].

Research suggests that miRNA occur within exosomes, not on their surface membranes or associated with surface structures [11,19]. Additionally, a significant number of transcripts in the exosomes are not present in the donor cells from which they are derived; this profiling suggests that the miRNA profile of exosomes does not directly reflect the transcriptional status of the donor cell [11,19]. Exosomes have therefore been proposed to be a means of cell type-specific intercellular paracrine communication through delivering RNAs, which would affect the recipient cell’s proteome, and this has been demonstrated in several in vitro models involving both animal and human cells [11,19,20,21,22,23].

While the principles of miRNA transfer by microvesicles are similar to that by exosomes, there are some notable differences [13]. For one, microvesicles (MVs) are synthesised from the plasma membranes of donor cells, and so the profile of the membrane proteins on them reflects the donor cell type. It seems probable that miRNA secretion via this mechanism is independent of the donor cell’s transcriptional status [24]. Known cell types that produce miRNA-loaded MVs include endothelial cells, mesenchymal stem cells, and cancer cells [12]. Finally, ECmiRNA are also found complexed with HDLs, and these have been of interest as biomarkers of CAD [25,26]. However, recent studies cast doubt on the exact role of exosomes and microvesicles as carriers of miRNA. One important criticism has been that extracellular vesicles may co-purify miRNA found in culture and supplement media, such as foetal bovine serum, potentially confounding results [27]. Newer techniques, such as high-resolution density gradient fractionation and direct immunoaffinity capture, suggest that the secretion of DNA and RNA products is independent of extracellular vesicles, perhaps through a proposed model of autophagy or multivesicular-endosome-dependent but exosome-independent mechanism [28]. Furthermore, only a small fraction of in vitro, human lymphocyte-derived extracellular vesicles have been found to carry miRNA, and the binding of extracellular vesicles to cell membranes has not been observed. This may be due to a short exposure time and variability in conditions from physiological conditions [29]. Thus, there is a requirement for further investigation in this domain, although microvesicle RNA biology has been successfully translated to use in clinical settings in the diagnosis of haematological and oncological disorders [30].

Freely-circulating miRNA have been demonstrated by PCR miRNA assays conducted on fractionated, filtered, and ultracentrifuged plasma obtained from peripheral blood samples [14,15]. The miRNA may be bound to Argonaute (Ago2), an extracellular miRNA binding protein, and together they form a stable nucleoprotein complex. These stable complexes exist intracellularly, so it may be plausible that a certain proportion of ECmiRNA are released following cell death processes, e.g., necrosis and apoptosis, though it remains a possibility that miRNA/Ago2 complexes are/can be directly released from cells in order to communicate with others [14,15].

## 3. Physiological Roles of miRNA and Their Clinical Relevance

The significance of miRNA is made evident by the defective organogenesis and embryonic lethality that is found in murine models of tissue-specific or germline Dicer knockouts, respectively [31,32]. Dysregulation of miRNA is linked to the aetiology or pathogenesis of viral infections, cancer, and metabolic diseases [33]. Notable cardiovascular examples are miR-208, miR-143/145, and miR-21. MiR-208 is derived from an intron of the α-MHC (myosin heavy chain) gene, which is uniquely expressed in the myocardium and encodes an isoform of myosin heavy chain [34]. It acts within a network to upregulate the expression of β-MHC in response to stress, but its absence does not result in the absence of myocardium, therefore yielding viable mice [35].

The miR-143/145 cluster regulates the expression of cytoskeletal genes in vascular smooth muscle cells (VSMCs), and although murine knockouts are still viable, they show reduced vascular tone and significantly reduced capacity for migration in the process of neointima formation following vascular injury [36,37]. Stress-induced hypertrophy of cardiomyocytes is, at least in part, facilitated by miR-21-mediated silencing of two target proteins [38]. Indeed, miRNA have a wide range of cardiovascular functions, and their absence induces many abnormal phenotypes [7]. A recently compiled database of extracellular vesicle miRNA describes their potential roles as biomarkers in various diseases, including myocardial infarctions [39,40,41,42].

Thus, miRNA may either have a causative role or are a consequence of pathology. In the case of the former, the relevant miRNA could be operating as an initiator or maintainer of the condition (i.e., is a necessary component of a particular disease process), or could simply yield susceptibility (i.e., could potentially be sufficient to produce the disease phenotype, for example by yielding a substrate that enables the disease to precipitate). In the case of the latter, the measured changes in miRNA levels may be due to unregulated secretion from injured/stressed cells, or as a homeostatic response to the insult, with the communication between the cells occurring at the paracrine or the endocrine level [43].

Hence, for both of these, by measuring the changes in the miRNA signatures in an individual before, during, and after recovery from a particular pathology, we may be able to identify how the miRNA are behaving with respect to aetiology and pathogenesis (i.e., whether changes in miRNA behaviour affect susceptibility, are an outright cause, or more simply are products of the disease process). These signatures themselves may be detected in biopsies or peripheral blood samples, and are defined by the identity of the specific miRNAs that are detected, as well as by their concentrations. There already exists diagnostic miRNA tests based on either an miRNA panel or single miRNA quantification for diseases like certain cancers, indicating a successful proof of concept for the use of miRNA as biomarkers in disease [44,45].

## 4. Coronary Artery Disease (CAD) Pathophysiology

To better appreciate and evaluate the potential of miRNA as biomarkers in CAD, it is necessary to first consider the pathology of CAD. The pathogenesis of coronary artery plaques in CAD involves endothelial cell activation and the subsequent infiltration of the tunica intima by oxidised lipoproteins and monocytes. These monocytes go on to differentiate into macrophages and transform into foam cells as they consume these lipoproteins [46,47]. Consequently, a chronic inflammatory response is produced, whereby the macrophages begin the secretion of cytokines and chemoattractant factors that promotes the activation of the endothelium, which beckons further adhesion and infiltration (diapedesis) of more monocytes/other leukocytes [48]. This leads to the development of a raised lesion with a fibrous cap (from the myofibroblasts) and a lipid-rich interior (from lysed foam cells). The “shoulder” of the cap is found to have both of these cell types in addition to T-lymphocytes (although their role is not entirely understood) [49].

Angiogenesis may occur within the plaque and contribute to the expansion of the plaque through haemorrhaging of the new vessels forming at the shoulder into the less dense, lipid-rich core. The plaque may subsequently rupture, which can lead to thrombosis as coagulation factors and thrombocytes adhere to the lesion, as well as embolisation of plaque fragments. Other complications include calcification, or the formation of an aneurysm as the tunica media weakens from the arterial remodelling [50]. Endothelial cells are also induced to produce a pro-inflammatory response, which propagates the further infiltration of monocytes and degeneration of the elastic laminae in the media [51]. This weakened area of vessel wall can dilate in response to pressure applied to it. CAD pathophysiology therefore has the potential to give rise to measurable circulating biomarkers, due to the close involvement of disease with the circulating vasculature. Established surrogates that are commonly used, such as circulating LDL, HDL, troponin, and creatinine kinase, are associated with different stages in their pathophysiology: LDL and HDL being more relevant upstream as risk factors, and troponin and creatinine kinase more relevant downstream as a consequence of sufficiently advanced disease. Thus, there may be other biomarkers that may be of use in either a prognostic or diagnostic capacity. miRNA may be one such class.

## 5. CAD Biomarkers and miRNA

Current standard molecular biomarkers include proteins, lipids, and other metabolites. Cardiac troponins are well-established and are commonly used indicators of adverse cardiac events [52]. Creatinine kinase is also used in the same context, although it is less specific due to its presence in skeletal muscle and cerebral tissue. However, troponins and creatinine kinase involve the terminal series of events in CAD: ischaemic damage to the myocardium arising as a result of acute coronary syndrome [9,53].

Novel serum biomarkers, and more recently urinary biomarkers for CAD, are of increasing interest. For example, high-sensitivity C-reactive protein and high-sensitivity troponin I assays have been proposed as biomarkers of coronary artery disease and its progression [54,55,56]. They pose the advantages of being non-invasive, compared to percutaneous coronary angiography, and without the radiation exposure of CT coronary angiography [57]. In a study that tested over 100 different serum biomarkers in over 1000 patients, four biomarkers in combination (adiponectin, apolipoprotein C-I, midkine, and kidney injury molecule-1 (KIM-1)) were found to predict incidence of severe CAD [58].

Alongside these novel biomarkers, numerous studies have tried to identify miRNA, which may distinguish between individuals with different cardiovascular health statuses (Table 1) [59,60,61]. These miRNAs may be considered at local sites, such as at plaques or sites of endothelial injury, or freely circulating in serum. There is a vast constellation of research, and several candidate miRNAs have been identified, although this is complicated by a lack of correlation between studies. This may be due to experimental design variation, as studies involve different experimental models, time courses (acute vs. chronic disease), and quantification methodologies.

### 5.1. Localised Changes in miRNA Profiles

**Figure 3:**
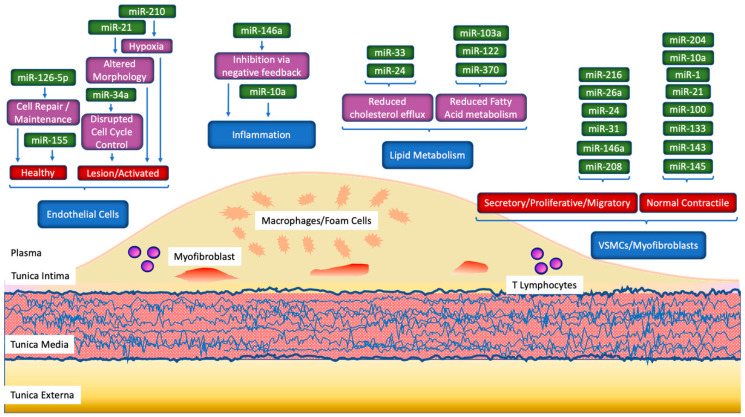
Associations between miRNA in different cells and constituent pathways in coronary artery disease (CAD) pathogenesis. Pathological alterations in the phenotypes of particular cells of the circulatory system, in addition to normal homeostatic processes, are core to the development of CAD (shown in blue, phenotypes in red, pathophysiological processes in purple). Various miRNAs have been identified as being associated with these pathological developments (shown in green), with previous studies showing that they may be implicated in particular contributing mechanisms. VSMC: vascular smooth muscle cell.

At the tissue level, specific miRNAs are expressed at the sites of myocardial injury/ischaemia or at the site of the atherosclerotic lesion. This expression may be in vascular tissue, myocardium, or plaque cells. Vessel wall biology changes drastically as atherogenesis progresses, and the changes in miRNA expression reflect this (Figure 3). In vascular smooth muscle cells. miRNA-1, -10a, -21, -100, -133, -143, -145, and -204 have been characterised with their standard contractile phenotype [62,63]. This is contrasted with a myofibroblast phenotype that VSMCs differentiate into during plaque development, which is instead associated with miRNA-24, -26a, -31, -146a, -208, and -221 [62,63]. The latter set of miRNA directs VSMCs to a secretory phenotype, with increased proliferative and migratory activity [63,64,65,66,67,68].

In the case of miRNA-21, however, there is evidence of the inverse, whereby this miRNA, which is elevated in CAD, may promote VSMC proliferation and indicate the progression of atherosclerosis [69]. Through the inhibition of FOXP1 and ZDHHC14 expression, respectively, miRNA-206 and -574-5p also act to do the same, and are demonstrated to be elevated in CAD patients, although the former seems to actually be anti-atherogenic [70].

Endothelial cells produce a baseline level of miRNA-155 and miRNA-126-5p under healthy conditions, whereas miRNA-21, miRNA-34a, and miRNA-210 are featured more in the endothelium of atherosclerotic lesions (Figure 3) [63]. These are due to increased shear stress from altered tissue morphology, interrupted cell cycle control, and hypoxia, respectively, all of which occur in atherosclerotic plaques. However, the downregulation of miRNA-126-5p removes a significant promoter of endothelial cell repair and maintenance, which further enables atherogenesis [71].

Endothelial progenitor cells (EPCs) are mobilised to give rise to endothelial cells in angiogenic atherosclerotic lesions, in a process marked by changes in the miRNA. In particular, miRNA-361-5p and miRNA-206, which are upregulated in CAD patients, are potentially responsible for controlling the expression of vascular endothelial growth factor in EPCs, as well as EPC activity [72,73]. Connections have been made with further miRNA, though only in the broad scope of these lesions as a whole (Table 1).

### 5.2. Changes in Circulating miRNA

Certain miRNA will be released from cells as either a homeostatic response to CAD or following cell death (Figure 3). Therefore, miRNAs that are linked to such insults, such as miRNA-499, miRNA-208, and miRNA-1 [74,75,76,77], could plausibly be released from ischaemic cardiomyocytes as they necrose. Muscle-enriched miR-133a, together with miR-1, shows a steeper and earlier increase than cardiac-enriched miRNA (miR-499 and miR-208b) upon myocardial injury [42]. Alternatively, cell death within the atherosclerotic lesion itself can produce circulating miRNA. A fraction of endothelial cells in atherosclerotic plaques undergo apoptosis, and thus release apoptotic bodies that have been found to contain miRNA-126 (Figure 3) [14]. In actuality, this miRNA acts through CXCL12 to stabilise plaques and protect the vessel wall structure from further damage under atherogenesis (Table 1).

In terms of homeostatic responses, leukocytes, such as peripheral blood mononuclear cells (PBMCs), demonstrate altered miRNA profiles in CAD patients relative to healthy controls. One study reported differences in the levels of miRNA-147, which was downregulated, and miRNA-135a, which was upregulated, in these cells [78]. Another group observed that CAD patients’ PBMCs also had an increased expression of miRNA-146a/b under inflammatory stimuli associated with CAD, and lowered expression of let-7i [79,80].

In another study comparing the expression levels of multiple circulating miRNAs between eight CAD patients and eight healthy volunteers, all of the miRNA primarily expressed in the endothelium—miRNA-126, -17, -20a, -92a, -221, -199a-5p, -27a, -130a, and -21, as well as let-7d—had significantly lower levels in the circulation in CAD patients [61] (note, however, that miRNA-126 is also highly enriched in platelets [81]). This was in contrast to the miRNAs that were specifically expressed in striated muscle, of which only one (miRNA-208b) was found to have a significant difference, and was instead elevated in CAD patients [61].

Determining the cell type of origin, as well as the precise roles of both of these types of miRNA, would further define the underlying communications and transformations that lead to plaque formation. However, it may be suggested that circulating miRNA are the most feasible candidates as biomarkers, due to the comparative ease of extraction

## 6. miRNA in CAD Pathophysiology

The relevance of miRNAs as biochemical precursors to the more macroscopic cellular and histological events that comprise atherogenesis is becoming increasingly evident [82,83]. As discussed above, lipid metabolism and inflammatory changes are key aspects of this process. Therefore, here we discuss miRNA in these contexts and highlight the changes that occur in pathological processes.

### 6.1. Lipid Metabolism

LDLs, mainly in their oxidised form, are the primary carriers of the cholesterol and triglycerides that are found in atherosclerotic lesions. Implicated in the synthesis of these molecules are miRNA-24, -33, -103a, and -122, all of which are found to have been significantly increased in the PBMCs of CAD patients [84,85]. Further investigations of miRNA-33 have reported that it suppresses the cholesterol efflux mechanism in cells, at least in part by inhibiting the expression of ATP-binding cassette transporter A1 [86,87]. Likewise, the expression miRNA-370 is also significantly raised in CAD patients [88]. This miRNA downregulates the expression of a carnitine palmitoyl transferase protein that is required for the trafficking of fatty acids into the mitochondria for β-oxidation, and is also higher in CAD patients [87]. In addition, these individuals can be identified from increased miRNA-486, -92a, -208a, -122, -93, and -17-5p [86,87].

### 6.2. Inflammation

Endothelial vulnerability to pathological inflammatory activity may, in part, be regulated by miRNA-10a, which is also reduced in CAD patients compared to healthy controls [71,82,89,90]. miRNA-155 shows the same trend, though there is contrasting evidence when the miRNA’s levels in the plasma and plaques of individuals with atherosclerosis were investigated [89,90]. Li et al. [90] have also shown that miRNA-155 reduces the expression of calcium-regulated heat stable protein 1 and promotes TNF-α expression in macrophages, suppressing foam cell formation [71,89,90].

Furthermore, miRNA-22 is known to repress the chemokine CCL2 in PBMCs, which modulates intercellular communication in inflamed tissues [82,91]. In CAD patients, the levels of these miRNAs in PBMCs are reduced. In addition to this, miRNA-146a is also reduced in CAD patients [91]. This miRNA is induced by pro-inflammatory cytokines to inhibit the nuclear factor-**κ**B pathway in a negative feedback loop to resolve inflammation in its later stages [82,91]. MiRNA, therefore, plays a role in inflammatory processes that may form one component of the complex pathophysiology of atherosclerosis, and may represent potential biomarker candidates early in the disease process [82].

## 7. Pitfalls in Assessing miRNA as Biomarker Targets

### 7.1. Confounding Factors

When considering the utility of miRNA as biomarkers, one needs to consider any variances in their expression not relating to pathological processes alone. One such source of variation may be population-level changes. Thus, studies has shown geographical/ethnic differences in the expression levels of miRNA [92,93].

Age and sex are other factors that correlate with the frequency of different miRNA in circulation [94]. This has been demonstrated in an analysis of platelet-derived mRNA and miRNA [95]. However, in terms of cardiac-specific miRNA, there is limited data on CAD-associated miRNAs and their variation with the sex and ethnicity of a patient. Discrepancies in miRNA levels with respect to age have been reported in a few studies, which is a further confounder, as ageing is a critical risk factor of cardiovascular health. For example, with miRNA-149, -424, and -765, the former two are downregulated and the latter upregulated in middle-aged (aged 49–57) CAD patients [96]. Another study showed that miRNA-126-3p expression is greater in senescent endothelial cells than in younger cells, and their quantity in circulation is also increased [97].

Furthermore, cardiac fibroblasts increase their expression of miRNA-21 and miRNA-22 with age, which can lead to increased fibrosis and progression towards senescence, respectively [98]. Cardiomyocytes show the same change in miRNA-22, whereby they have been demonstrated to have a suppressive effect on autophagy in aged cardiomyocytes, producing an improved functional recovery of myocardium post-infarct in elderly mice, though not in younger mice [99].

Moreover, the level of miRNA-155, in addition to actually being higher in human females, decreases with age. Other miRNA have been implicated in cardiac ageing and associated dysfunction in addition to CAD, including the miRNA-17-92 cluster, miRNA-18, miRNA-19, and miRNA-17-3p [98,100,101,102]. Therefore, since not all studies investigating these miRNA have adjusted their results to control for these factors, any reported variation in miRNA may partially be explained by factors other than CAD [103].

### 7.2. Measuring Serum and Plasma miRNA

Of the published studies that analyse circulating miRNA, the general trend seems to concentrate on using plasma-based samples. This specification is critical, as the difference in the molecular profile between serum and plasma results in a difference between the recorded miRNA levels as the sample is being prepared, as serum holds a higher concentration of RNA than plasma [104]. Further to this point is that coagulation increases variability in serum miRNA concentrations [104]. Hence, we must recognise the difficulty of normalising miRNA values due to pre-analytical variations, including blood cell counts and the miRNA load of the cells and platelets in circulation.

Furthermore, any haemolysis releasing the miRNA contained within blood cells will affect the total miRNA concentration and profile that we identify from serum, though cellular contamination would cause the same changes in plasma and serum samples. Thus, care should be taken when preparing samples to prevent distorted results [104,105,106], and it may be best to produce a standard operating procedure (SOP) for acquiring miRNA data, based on currently existing SOPs for collecting such samples.

## 8. Validity of miRNA as Biomarkers

When addressing the feasibility/validity of miRNA as a biomarker, a few critical points must first be considered. Firstly, ease of access is not a concern, as miRNAs occur in peripheral blood so whole blood samples can be taken. However, it should be noted that the majority of miRNA in peripheral blood will likely be derived primarily from well-vascularised tissues, e.g., lungs and kidneys, in addition to blood cells themselves (platelets are a major contributor to the circulating RNA pool [107]), so the relative quantities of particular miRNAs should be taken into account.

Secondly, cost is likely a significant concern, due to the processes required to prepare the miRNA: RNA purification, reverse transcription–polymerase and quantitative polymerase chain reaction/microarrays/sequencing, controlling RNase activity, etc. [108,109]

Thirdly is timing/storage. MiRNA/Ago2 complexes have remarkable biologic stability and occur both in microvesicles and freely in plasma, though miRNA integrity is also maintained in tissues that have been fixed in formalin and embedded in paraffin, as is done with biopsies [108,109]. This protects the original samples, though preparation of purified miRNA must still be done carefully, and regular monitoring and appropriate storage are necessary. Control of the temperature and RNase activity are crucial to prevent degradation.

Lastly is content/criterion validity [110]. Major efforts have been and are currently being invested into establishing the reliability of miRNA as a diagnostic for a diverse range of human diseases (i.e., carrying indicative or predictive value), as well as into developing diagnostic tests for them and trying to understand their contribution to the disease’s manifestation, as mentioned [43,111,112,113,114]. Prognostic information and severity assessments also stand to be improved through the use of miRNA [115].

While the quantification and normalisation methodologies are still being developed, stability, accessibility, and disease specificity still lend miRNAs significant value as biomarkers, and evidence of this continues to grow [116,117].

Given that there are several miRNAs, it is likely to be beneficial to assay these particular biomarkers in a panel of tests. When considering the levels of all of those that are tested for, we gain a better understanding of the pathology’s context. Some groups support this notion, with the suggestion that using a panel of select miRNA “may have a greater target-organ specificity and better diagnostic value than a single miRNA or well-established clinical biomarker” [116]. This is easily demonstrated by the range of miRNAs that are found to be involved in a singular disease, and a singular miRNA may be involved in multiple diseases, producing a web of interaction [116]. A gene can have sequences complementary to different miRNA seed sequences, and an miRNA may target multiple genes, so this is a feasible paradigm.

## 9. Conclusions

The field of miRNA biomarkers is still relatively young, although it shows significant promise for diagnostics, including for CAD. Many candidate biomarkers have been investigated which characterise different aspects of this vascular disease. There are still challenges, both in the scientific understanding of their roles in CAD and in normalising their measured values across samples and accounting for natural variation in the healthy population.

## Figures and Tables

**Table 1:** miRNAs as active factors/potential biomarkers in CAD and associated pathologies. Methodologies of miRNA identification, quantification, sample location, experimental model, and time course of disease pathology are indicated. Where the quoted reference is a review article synthesizing several sources of evidence, this has been indicated.

References	miRNA	Quantitative Effect	Outcome	Sample Type	miRNA Identification/Quantification Method	Cell Lines/Study Population	Acute/Chronic Disease Status
Wang et al., 2016 [118]	miRNA-146a	Upregulated	This miRNA may be a potential biomarker for poor coronary collateral circulation in CAD patients.	Plasma	qRT-PCR	Human patients	Chronic (1-month cut-off)
Li et al., 2017 [119]	miRNA-155-5p miRNA-483-5p miRNA-451a	MiRNA-155-5p and miRNA-483-5p are upregulated; miRNA-451a is down-regulated	Potential biomarkers for the early detection of atherosclerotic plaque rupture.	Plasma	qRT-PCR	Human patients	Stable CAD
Zhao et al., 2015 [37]	miRNA-143 miRNA-145	Contested	Altered in CAD. Potentially released from vascular walls.	Plasma	(Review article)	(Review article)	(Review article)
Li et al., 2017 [120]	miRNA-122 miRNA-140-3p miRNA-720 miRNA-2861 miRNA-3149	Upregulated	Elevated during the early stages of ACS.	Plasma	qRT-PCR	Bama male minipigs and human patients	Minipigs: normal and acute MI. Human patients: Stable angina, unstable angina and acute MI.
Jansen et al., 2017 [121]	miRNA-21 miRNA-126-3p miRNA-222	Upregulated	These miRNAs increased in concentration following periods of cardiac stress in patients with stenosed coronary arteries.	Plasma	qRT-PCR	Human patients	Stable CAD
Soeki et al., 2015 [122]	miRNA-100	-	Associated with coronary plaque instability. Potentially released from plaques.	Plasma	qRT-PCR	Human patients	Unknown
Liu et al., 2017 [123]	miRNA-29a	Upregulated	Moderates expression of mRNAs of extracellular matrix proteins. Associated with atherosclerosis and intima-media thickness of carotid arteries.	Plasma	qRT-PCR	Human patients	Unknown
Wang et al., 2017 [124]	miRNA-126	Downregulated	A potential biomarker for CAD. Inversely correlated to placenta growth factor.	Plasma	qRT-PCR	Human patients	CAD for 15–24 months
Al-Kafaji et al., 2017 [125]	miRNA-126	Downregulated	A potential biomarker for CAD. Inversely correlated with LDL concentration.	Plasma	qRT-PCR	Human patients	Type 2 diabetics, some with CAD diagnoses
Al-Muhtaresh et al., 2019 [126]	miRNA-1 miRNA-133	Upregulated	Potential biomarkers. Both correlate with LDL-C levels; miR-1 is known to negatively regulate Bcl2 [127].	Plasma	qRT-PCR	Human patients	Type 2 diabetics, some with CAD diagnoses
Zernecke et al., 2009 [14]	miRNA-126	-	Released from apoptotic bodies derived from endothelial cells from atherosclerotic plaques. Reduces inflammatory activity/plaque development.	Plasma/Plaque	qRT-PCR	Human aortic smooth muscle cell culture. Human atherosclerotic plaques. ApoE^−/−^ murine endothelial cell cultures. HUVEC cell line	Unknown
Wang et al., 2014 [128]	miRNA-31 miRNA-720	Downregulated	Potential biomarkers for early CAD.	Plasma/endothelial progenitor cells	qRT-PCR	Human patients	Unknown CAD
Zhang et al., 2017 [129]	miRNA-208a	-	Significant association with Gensini score, and by extension the severity of atherosclerosis. Potential biomarker for CAD severity.	Plasma	qRT-PCR	Human patients	Unknown CAD
Jansen et al., 2014 [111]	miRNA-126 miRNA-199a	-	The levels of these miRNA, which occur in circulating microvesicles, are potentially prognostic for major adverse cardiovascular events in patients with stable CAD.	Plasma	qRT-PCR	Human patients	Stable CAD
Han et al., 2015 [130]	miRNA-21 miRNA-23a miRNA-30a miRNA-34a miRNA-106b	Upregulated	These miRNAs occur at higher levels in ApoE^−/−^ mice, which models hypercholesterolaemia. MiRNA-21, -23a, and -34a are potential biomarkers for CAD. MiRNA-21 has been linked to CAD-derived ACS.	Plasma	qRT-PCR and miRNA microarrays	ApoE^−/−^ mice and human CAD patients	Unknown
Zhou et al., 2016 [70]	miRNA-206 miRNA-564-5p	Upregulated	Potential biomarkers for CAD	Plasma	qRT-PCR and miRNA microarrays	Human patients	Unknown
Sayed et al., 2015 [96]	miRNA-149 miRNA-424 miRNA-765	MiRNA-149 and miRNA-424 were upregulated, miRNA-765 was downregulated	Potential biomarkers for CAD in middle-aged patients	Plasma	qRT-PCR	Human patients	Stable and unstable CAD
Gao et al., 2015 [131]	miRNA-145	Downregulated	This miRNA regulates VSMC fate, inhibiting proliferation. It is the modal miRNA in healthy vessel walls, though in atherosclerotic plaques it may not even be detected. Plasma concentration levels are significantly reduced in CAD patients, and those with three-vessel disease have a significantly lower quantity as well. Potential biomarker for CAD.	Plasma/plaque	qRT-PCR	Human patients	Unknown (patients diagnosed with CAD for more than a year)
Ren et al., 2013 [132]	miRNA-106b/25 cluster miRNA-17/92a cluster miRNA-21/590-5p cluster miRNA-126 miRNA-451	Upregulated in patients with unstable angina, though there is evidence that miRNA-17/92a was actually downregulated in CAD patients [83]	These miRNAs are elevated in CAD patients relative to those with stable AP. MiRNA-17/92a is involved in angiogenesis, which further complicates plaques. Increased miRNA-21 can yield increased MMP activity, which can hinder plaque progression. Potential biomarkers for CAD.	Plasma	qRT-PCR	Human patients	CAD and unstable angina
Chen et al., 2015 [133]	miRNA-17-5p	Upregulated	Potential biomarker for early CAD.	Plasma	qRT-PCR	Human patients	Unknown
Faccini et al., 2017 [89]	miRNA-155 miRNA-145 let-7c	Downregulated	Potential biomarkers for CAD	Plasma	qRT-PCR and miRNA microarrays	Human patients	Unknown
Koroleva et al., 2017 [51]	miRNA-21 miRNA-100 miRNA-127 miRNA-133 miRNA-143/145 miRNA-221/222 miRNA-494	All upregulated apart from miRNA-221/222, which was downregulated	The expression of these miRNA may influence plaque stability: miRNA-21, -143, and -221 are pro-stability; miRNA-100, -127, -133, and -494 are pro-instability.	Plaque	(Review article)	(Review article)	(Review article)
Lin et al., 2016 [134]	miRNA-365	Downregulated	Regulation of the inflammatory response, specifically IL-6 activity, such that IL-6 expression increases as miRNA-365 expression decreases.	Plaque, serum, and circulating monocytes	qRT-PCR	Human patients	Unknown (patients with atherosclerosis)
Cipollone et al., 2011 [135]	miRNA-100 miRNA-127 miRNA-145 miRNA-133a/b	Upregulated	The expression of these miRNA varies with plaque stability. MiRNA-133 is relevant to stroke-related proteins and is thought to be vascular smooth muscle-specific.	Plaque	qRT-PCR	Human patients	Unknown
Kumar et al., 2014 [136]	miRNA-712 miRNA-205	Upregulated in atherosclerosis	These miRNA target and reduce expression of metalloproteinase inhibitor 3 (TIMP3), increasing the activity of matrix metalloproteinases (MMPs), which affects inflammatory processes and VSMC/leukocyte migration in atherosclerosis.	Endothelial cells (Plaque)	Review (qRT-PCR, microarrays, and fluorescent in situ hybridisation)	Review (mice (C57BL/6 and ApoE^−/−^))	Review (unknown)
Tian et al., 2014 [137]	miRNA-155	Upregulated	Raised inflammatory response and foam cell differentiation.	Monocytes (plaque)	qRT-PCR	ApoE^−/−^ mice	Unknown
Horie et al., 2012 [138]	miRNA-33	-	Deficiency in ApoE knockout mice suppressed atherogenesis/plaque progression.	Monocytes/macrophages (plaque)	qRT-PCR	ApoE^−/−^ mice	Unknown
Fang et al., 2010 [139]	miRNA-10a	Downregulated	Expression levels were reduced in endothelial cells that are thought to be pre-atherosclerotic, affecting inflammation signalling.	Endothelial cells (plaque)	qRT-PCR, miRNA microarrays, and fluorescent in situ hybridisation	Adult pigs	Unknown
Zernecke et al., 2009 [14]	miRNA-126	-	Released from apoptotic bodies derived from endothelial cells from atherosclerotic plaques. MiRNAs reduce inflammatory activity/plaque development.	Plasma/plaque	qRT-PCR	Human aortic smooth muscle cell culture. Human atherosclerotic plaques. ApoE^−/−^ murine endothelial cell cultures. HUVEC cell line.	Unknown
Raitoharju et al., 2011 [62]	miRNA-21 miRNA-34a miRNA-146a miRNA-146b-5p miRNA-210	Upregulated	These miRNAs were upregulated in plaques compared to left internal thoracic arteries that were not atherosclerotic. This has been linked to VSMC changes seen in atherogenesis.	Plaque	miRNA microarrays and qRT-PCR	Human patients	Unknown
Shan et al., 2015 [140]	miRNA-223	Upregulated	This miRNAs seems to be secreted from cells in the circulation. Their levels are elevated in the serum and atherosclerotic lesions in apolipoprotein-E knockout mice.	Plaque serum/blood cells	qRT-PCR	Sprague–Dawley rat VSMC cultures and C67BL/6 murine platelets	Unknown
Bidzhekov et al., 2012 [141]	miRNA-26b miRNA30e-5p miRNA-105 miRNA125a-5p miRNA-520b	MiRNA-26b, -30e-5p, and -125a-5p were upregulated. MiRNA-105 and miRNA-520b were downregulated.	These miRNAs had altered expression in CAD patients relative to healthy controls.	Plaque, monocytes	qRT-PCR and miRNA microarrays	Human patients	Unknown
Jansen et al., 2013 [142]	miRNA-126	Downregulated	Circulating levels of miRNA-126 decreased in CAD patients.	Circulating microparticles	qRT-PCR	Mice and human patients	Stable CAD since 2003
Schulte et al., 2015 [143]	miRNA-197 miRNA-223	-	Strong prognostic value in CAD patients for cardiac death.	Serum	qRT-PCR	Human patients	Unknown CAD
Hulsmans et al., 2012 [144]	miRNA-181a	Downregulated	Potential biomarker for CAD, as well as metabolic syndrome	Monocytes	qRT-PCR and miRNA microarrays	Human patients	Unknown

ACS: acute coronary syndrome, ApoE: Apolipoprotein E, CAD: coronary artery disease, HUVEC: human umbilical vein endothelial cells, MI: myocardial infarction, qRT-PCR: quantitative real time polymerase chain reaction.
